# A Diagram to Define the Lives of the Patriarchs, and the Early History of the Seed of the Serpent and the Seed of the Woman, Particularly in Reference to the Origin of Disease and the Danger of Unsanctified Knowledge

**Published:** 1843-10

**Authors:** 


ART. VI.—*A Diagram to define the Lives of the Patriarchs, and the early History of the Seed of the Serpent and the Seed of the Woman, particularly in reference to the origin of disease and the danger of unsanctified knowledge.* By H. L. SMITH, M.R.C.S. &c.—*Cheltenham*, 1842.

RELIGIOUS physic seems to be fashionable. Our only wish, for the sake of religion as well as physic, is that the authors of works of this kind intermingled a little more common sense with their piety.

The writer of this odd little work seems in character to be the converse of that prince of whom we are told, "he never said a foolish thing, and never did a wise one." Judging from his book only, we should set him down as a man who was theoretically a visionary; but actually a benevolent, useful, well-principled, and charitable man. Spinning upon paper the thinnest and most unsubstantial theories; drawing large conclusions from the slenderest premises; generalizing from a single and a very doubtful observation; vain, self-opinionated, dogmatic, but with right and sound principles of duty; sincere, in earnest, actively benevolent to his poorer fellow-men; always right-hearted, however wrong-headed. The bulk of the volume is a religious theory; sent to the editor, we presume, from its containing an explanation of the "origin of disease;" and with this it is our province alone to deal.—Before the flood the earth was watered by a mist, not by rain; and the patriarchs lived on an average 912 years. But the flood came, and the earth was for a year saturated with moisture; and the average of the life of the post-diluvial patriarchs was only 332 years. Such is Mr. Smith's statement of facts; and the conclusion he draws is, that as wet is injurious to health the dampness of the earth, from its submersion for a whole year and from rain subsequently, was the cause of this shortening of life! But although Mr. Smith quotes the Bible copiously he has not read it attentively, for we are distinctly told that *before* the flood God shortened man's life to 120 years (Genesis, vi. 3,) so that this theory of wet is good for nothing.—There is, says Mr. Smith, another cause, an internal one, and this is anger, "a general term which implies many others; by some it is called passion, choler, irritability." (p. 27.) Recollecting that the world was destroyed by the flood on account of its being "corrupt and filled with violence," we felt curious to know on what grounds it could be asserted that we must explain our shortened span through the medium of so common an infirmity. And the only evidence brought forward to ground such a broad statement is "the fact (as recorded by Moses, and clearly shown in the diagram,) that Nahor,—the simple translation of whose name is 'Angry,'—and who was the eighth patriarch from the flood, *lived the shortest time* of any of the first ten post-diluvial patriarchs, being only 148 years of age when he died." (p. 27.) An exquisite example of a crotchet leading common sense astray!

Besides this lack of judgment and this marvellous defect in the reasoning powers, there is observable in the little book many indications of a vanity which is at once pitiable and ludicrous. This leads Mr. Smith to congratulate himself on his own deficiency of scientific knowledge: "I have digressed,—I may be wrong—I confess to being *willingly* unskilled in political economy, to being also *willingly* unskilled in the use of the geological hammer," &c. This, and much like this in the same strain, shows that if, like Dr. Johnson's opponent, Mr. Smith thanks God for his ignorance, he has very much to be thankful for. But, after such debility and puerility it is refreshing to reach the appendix, and to find the theoretical writer to be a practically benevolent man; originating the self-supporting dispensaries, taking pains to carry into effect in his own neighbourhood the allotment system, and executing kind-hearted schemes for the amusement of the poor. Engaged in such active duties we respect him and wish him well; but he should leave "the patriarchs" to theologians, and avoid theorising.

